# Hantavirus Infections among Overnight Visitors to Yosemite National Park, California, USA, 2012

**DOI:** 10.3201/eid2003.131581

**Published:** 2014-03

**Authors:** Jonathan J. Núñez, Curtis L. Fritz, Barbara Knust, Danielle Buttke, Barryett Enge, Mark G. Novak, Vicki Kramer, Lynda Osadebe, Sharon Messenger, César G. Albariño, Ute Ströher, Michael Niemela, Brian R. Amman, David Wong, Craig R. Manning, Stuart T. Nichol, Pierre E. Rollin, Dongxiang Xia, James P. Watt, Duc J. Vugia, for the Yosemite Hantavirus Outbreak Investigation Team

**Affiliations:** California Department of Public Health, Richmond and Sacramento, California, USA (J.J. Núñez, C.L. Fritz, B. Enge, M.G. Novak, V. Kramer, S. Messenger, M. Niemela, D. Xia, J.P. Watt, D.J. Vugia);; Centers for Disease Control and Prevention, Atlanta, Georgia, USA (J.J. Núñez, B. Knust, L. Osadebe, C.G. Albariño, U. Ströher, B.R. Amman, C.R. Manning, S.T. Nichol, P.E. Rollin);; National Park Service, Fort Collins, Colorado, and Albuquerque, New Mexico, USA (D. Buttke, D. Wong)

**Keywords:** hantavirus, hantavirus pulmonary syndrome, Sin Nombre virus, Peromyscus spp., respiratory infections, viruses

## Abstract

In summer 2012, an outbreak of hantavirus infections occurred among overnight visitors to Yosemite National Park in California, USA. An investigation encompassing clinical, epidemiologic, laboratory, and environmental factors identified 10 cases among residents of 3 states. Eight case-patients experienced hantavirus pulmonary syndrome, of whom 5 required intensive care with ventilatory support and 3 died. Staying overnight in a signature tent cabin (9 case-patients) was significantly associated with becoming infected with hantavirus (p<0.001). Rodent nests and tunnels were observed in the foam insulation of the cabin walls. Rodent trapping in the implicated area resulted in high trap success rate (51%), and antibodies reactive to Sin Nombre virus were detected in 10 (14%) of 73 captured deer mice. All signature tent cabins were closed and subsequently dismantled. Continuous public awareness and rodent control and exclusion are key measures in minimizing the risk for hantavirus infection in areas inhabited by deer mice.

## Abstract 2

TOC summary: A rare hantavirus outbreak reaffirms the need for control of deer mice and public awareness of the risks posed by contact with them.

Hantavirus pulmonary syndrome (HPS) is an acute viral disease, characterized by a nonspecific febrile illness followed by severe noncardiogenic pulmonary edema and cardiogenic shock. It is also referred to as hantavirus cardiopulmonary syndrome. Approximately 36% of reported HPS cases in the United States are fatal (*1*). HPS was first recognized in 1993 when an outbreak of unexplained respiratory deaths occurred among otherwise healthy adults in the southwestern United States (*2*–*5*). A previously unknown hantavirus, Sin Nombre virus (SNV), was identified as the etiologic agent (*6*,*7*). Deer mice (*Peromyscus maniculatus*) are the reservoir for SNV (*8*), which infected mice shed in their urine, saliva, and feces. Humans are exposed chiefly by inhaling aerosolized excreta. Activities associated with increased risk for HPS include occupying or cleaning rodent-infested buildings, sweeping or disturbing rodent excreta or nests, sleeping on the ground, and handling mice without gloves (*9*–*11*). Person-to-person transmission of SNV has not been documented (*12*).

During 1993–2011, a total of 587 cases of HPS were confirmed in the United States, primarily in western states (Centers for Disease Control and Prevention [CDC]/Viral Special Pathogens Branch, unpub. data). Although household clusters of HPS have been reported (*13*,*14*), most HPS cases occur as single, sporadic disease incidents. In August 2012, the California Department of Public Health (CDPH) confirmed HPS in 2 California residents, both of whom had visited Yosemite National Park (Yosemite) in June 2012 and had lodged in so-called signature tent cabins (which differ from regular tent cabins by having an interior wall and roof consisting of drywall with a layer of foam insulation between the drywall and exterior canvas) in the Curry Village area of Yosemite Valley. Because of the common travel history of these otherwise nonepidemiologically unrelated patients, CDPH, in collaboration with CDC, the National Park Service (NPS) Office of Public Health, and others, initiated an investigation of a possible HPS outbreak associated with Yosemite. This report summarizes the clinical, epidemiologic, laboratory, and environmental findings of the investigation.

## Methods

### Epidemiologic and Laboratory Investigation

We defined a case-patient as a person who had 1) illness onset June 1–October 31, 2012, clinically compatible with hantavirus disease; 2) laboratory confirmation of SNV infection by detection of IgM against SNV or increasing titers of IgG in serum, SNV-specific antigen shown by immunohistochemical test (IHC), or SNV-specific nucleic acid sequences by reverse transcription PCR (RT-PCR); and 3) a history of visiting Yosemite during the 6 weeks preceding illness onset. HPS was defined as fever ≥38.3**°**C with bilateral diffuse interstitial edema and respiratory compromise within 72 hours of hospitalization, occurring in a previously healthy person, or an unexplained respiratory illness resulting in death, for which an autopsy demonstrated noncardiogenic pulmonary edema without an identifiable cause (*15*).

Finding case-patients was facilitated through health alerts issued by CDPH, NPS, and CDC to health care providers, advising them to contact state or local public health officials to obtain confirmatory testing of patients with suspected HPS who had visited Yosemite. Commercial diagnostic laboratories that detected hantavirus antibody in submitted serum specimens were asked to notify and forward specimens to state public health laboratories or CDC for confirmatory testing. State health departments that identified the death of a previously healthy person who had a history of travel to Yosemite were asked to collect tissue specimens for confirmatory testing by CDC. CDPH and NPS issued media releases recommending that persons who experienced febrile illness after travel to Yosemite consult with their health care provider about possible hantavirus infection. Notifications were sent to all visitors registered as overnight guests at Yosemite during June 1–September 12, 2012, advising them to seek medical attention if they experienced symptoms of hantavirus infection.

Acute- or convalescent-phase serum specimens were tested at CDPH’s Viral and Rickettsial Diseases Laboratory or CDC’s Viral Special Pathogens Laboratory for IgM and IgG against SNV by using the same ELISA (*7*). Four-fold dilutions were performed (1:100–1:6400), and titers ≥400 were considered positive. Formalin-fixed tissues from patients who died were tested for the presence of hantavirus antigen by IHC at CDC’s Infectious Diseases Pathology Branch (*16*). A nested RT-PCR (*17*) was performed by using RNA extracted from frozen tissue. A TaqMan assay (Roche Molecular Systems, Inc., Pleasanton, CA, USA), targeting the small (S) segment RNA from North American hantaviruses, was used on RNA samples extracted from fixed tissues. This assay was used following standard protocols (SuperScript III Platinum One-Step qRT-PCR Kit w/ROX; Invitrogen, Carlsbad, CA, USA) with the following primers and probes: 143F 5′-TGGACCCIGATGAYGTTAACAA-3′, 303R 5′-ATCAGGITCAAKCCCIGTTGG-3′, and NA-181P 5′-FAM-AGACGGGCIGCTGTGTCTGCATT-BQH-3′. Medical records for laboratory-confirmed HPS patients were reviewed for relevant clinical information.

Patients with confirmed cases (or a proxy family member if the patient was deceased) and their non-ill travel companions who had shared lodging with the patient were interviewed by telephone, using a standardized questionnaire. One non-ill companion was interviewed for each patient identified. By using closed and open-ended questions, patients and non-ill companions were asked about demographic characteristics, illness history, travel, and other activities in the weeks preceding illness onset. Participants were asked about the type of lodging and activities they experienced during their Yosemite visit, particularly actions (e.g., cleaning or sweeping), observations (e.g., mouse droppings), and behavior (e.g., sleeping on the floor) potentially associated with exposure to rodents and SNV. Data from questionnaires were maintained in a spreadsheet by using standard software (Excel; Microsoft, Redmond, WA, USA). The incidence of SNV infection among guests staying in a signature tent cabin was compared with that of guests staying in other lodgings in Curry Village, according to Yosemite guest registry records for June 1–August 28, 2012. A difference was considered statistically significant if p≤0.05.

### Environmental Investigation

Yosemite lodgings where patients had stayed and other nearby buildings were visually evaluated for evidence of active or recent rodent activity (e.g., nesting materials, feces, or urine stains) as well as structural features that might allow rodent entry. Rodent populations in and around Yosemite guest lodgings were live-trapped for evaluation. Sherman live-traps (H.B. Sherman Traps, Tallahassee, FL, USA) were baited with rolled oats and placed overnight inside and outside buildings. Rodent abundance was estimated by trap success, defined as the ratio of captured mice to the total number of traps set. Captured rodents were anesthetized, euthanized, measured, and identified to species. A minimum of 11 μL of blood was collected from the retrobulbar sinus of each mouse and sent to CDPH for ELISA testing for IgG against SNV.

## Results

### Laboratory, Clinical, and Epidemiologic Findings

Ten case-patients were identified among persons who had visited Yosemite overnight during summer 2012. The median age of the 10 patients was 44.5 years (range 12–56 years). Eight were residents of California, 1 of Pennsylvania, and 1 of West Virginia. Infection was confirmed by serologic testing for 8 patients and by IHC and RT-PCR for 2 patients. A 540-bp fragment amplified from fresh frozen tissues of 1 case-patient who died had high homology (>90%–99% identity) with the known SNV isolates Convict Creek, NM R11, and NM H10 (GenBank accession nos. AF425256.1, L37902.1, and L37901.1, respectively).

Salient case-patient clinical characteristics are displayed in the Table. Eight patients had respiratory signs or findings compatible with HPS, 3 of whom died. Seven HPS case-patients were hospitalized for a median of 7 days (range 2–30 days); 5 required intensive-level care with ventilatory support. 

**Table:** Clinical characteristics of 10 hantavirus case-patients who were exposed at Yosemite National Park, 2012

Patient characteristic	No. affected
Male sex	6
Illness clinically compatible with hantavirus pulmonary syndrome	8
Hospitalized	7
Intubated	5
Died	3
Pulmonary edema on chest radiographs	7*
Thrombocytopenia (<150,000/mm^3^)	8*
Elevated hematocrit (>50%)	1*
Elevated creatinine (>1.3 mg/dL)	4*


*Diagnostic tests were not performed for 2 case-patients, 1 with only mild symptoms and 1 who died.

The illnesses of 2 case-patients did not fulfill the complete HPS definition. One patient had been examined in an emergency department for fever, cough, and shortness of breath and was discharged and recovered. That patient’s chest radiograph and computed tomographic scan revealed no evidence of pulmonary infiltrates or edema. The second patient did not report fever or pulmonary symptoms but had severe headache, nausea, vomiting, and diarrhea. Both patients were tested for SNV retrospectively after they heard about the Yosemite outbreak in the news and had detectable antibodies against SNV.

**Figure 1:**
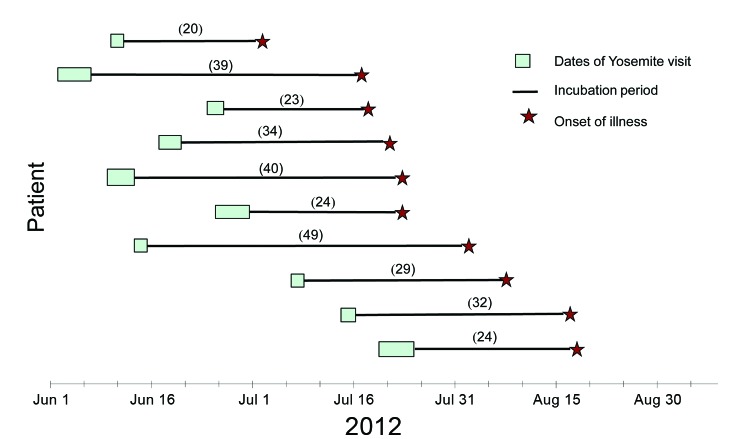
Dates of stay in Yosemite National Park, incubation periods, and dates of illness onset for 10 case-patients, 2012. Incubation period calculated as days from last date of Yosemite stay to first date of illness.

The onset of illness for the 10 case-patients occurred from July 2 through August 16, 2012 (Figure 1). They had stayed overnight at Yosemite during June 3–July 23, 2012; the median incubation period was 30.5 days (range 20–49 days). From interviews with case-patients (or their proxy and non-ill companions), we found that case-patients reported engaging in activities and behavior similar to those of non-ill companions while at Yosemite, and no single activity typically associated with risk for HPS was common among patients.

Nine case-patients had lodged 1–6 nights in a signature tent cabin in the Boystown area of Curry Village. One of these patients stayed in the same cabin in which another patient had lodged 13 days earlier. Staying overnight in a signature tent cabin was significantly associated with SNV infection: 9 of 10,193 guests who registered to stay in a signature tent cabin during June 1–August 28, 2012, had confirmed SNV infection, compared with 0 of 40,288 registered guests of other cabins in Curry Village during the same period (p<0.001). One case-patient had not lodged in Curry Village but had stayed in 4 different regular tent cabins in the Tuolumne Meadows area of Yosemite, ≈16 miles northeast of Curry Village and >4,700 feet higher in elevation.

Of all guests who registered to stay in a signature tent cabin during this time, ≈50% were from California, 30% were from other states, and 20% were from other countries. No SNV infections were reported among international visitors to Yosemite. No hantavirus infections were reported among ≈2,800 NPS and contract employees who worked in Yosemite during the outbreak period. 

### Environmental Findings

**Figure 2:**
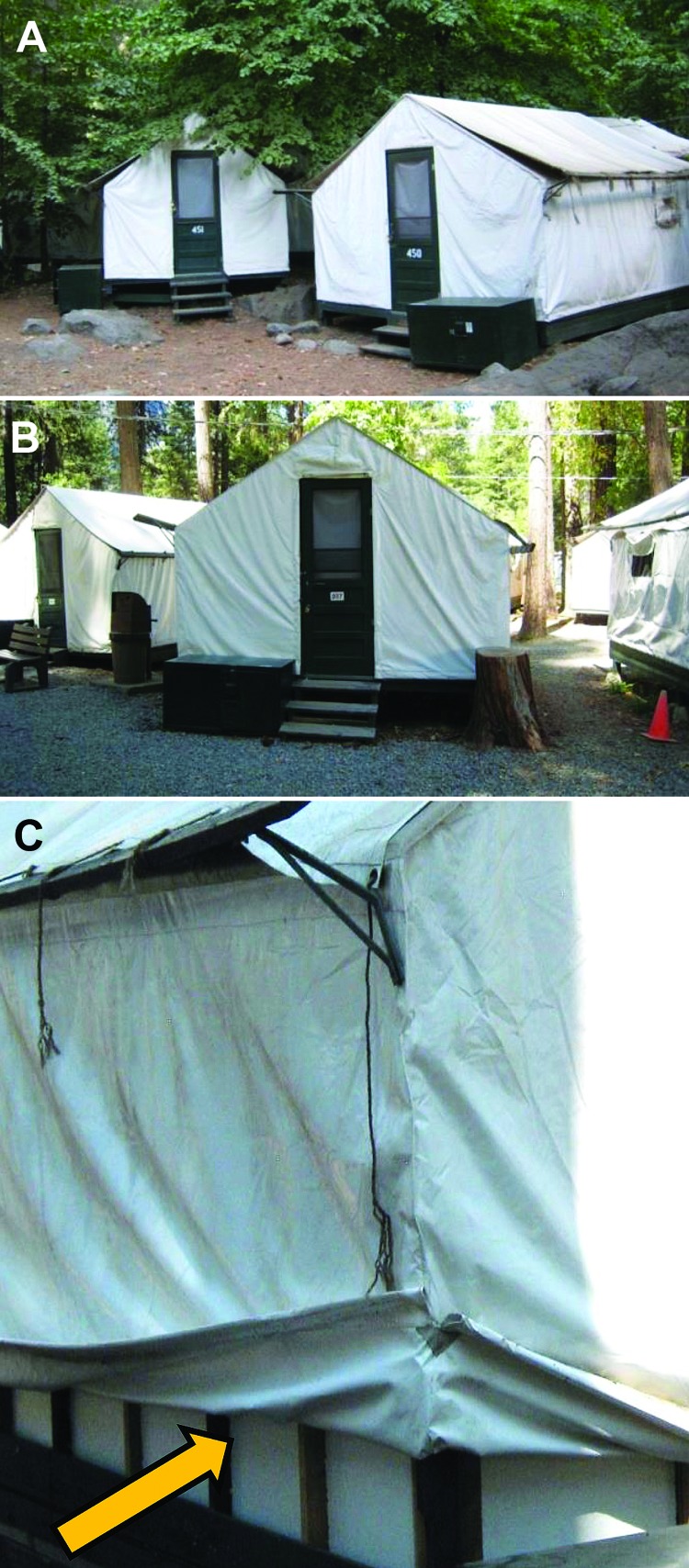
Regular and signature tent cabins, Yosemite National Park, summer, 2012. A) Outside view of a regular tent cabin. B). Outside view of a signature tent cabin. C) Inner layer of foam insulation under the canvas of a signature tent cabin.

**Figure 3:**
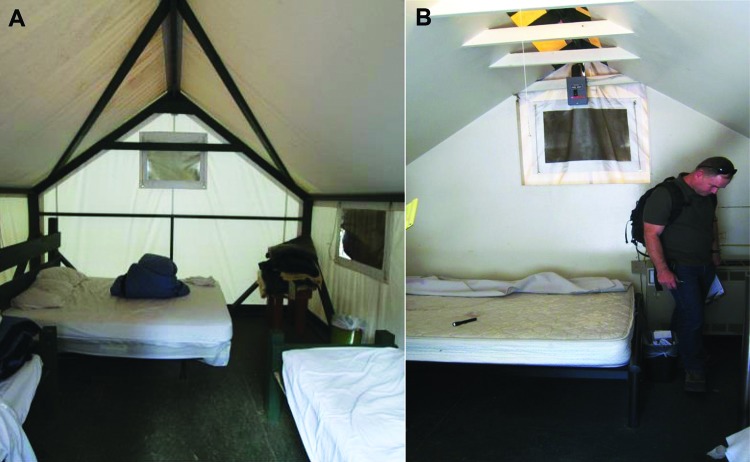
Inside view of regular and signature tent cabins, Yosemite National Park, summer 2012. A) Regular tent cabin showing canvas affixed over a wood frame. B) Signature tent cabin showing drywall between the exterior canvas and the interior living space.

Guest lodgings in Curry Village, at an elevation of ≈4,000 feet above sea level, consisted of 319 regular tent cabins, 91 signature tent cabins, 18 standard hotel rooms, and 70 hard-sided wood cabins on ≈23 acres. All signature tent cabins were located in the single 2.5-acre Boystown part of Curry Village. Both regular tent cabins and signature tent cabins are constructed of an exterior heavy canvas affixed over a wood frame elevated off the ground on a wood base (Figures 2, panels A, B). The distinct characteristics of signature tent cabins are shown in Figure 2, panel C, and Figure 3, panel B.

**Figure 4:**
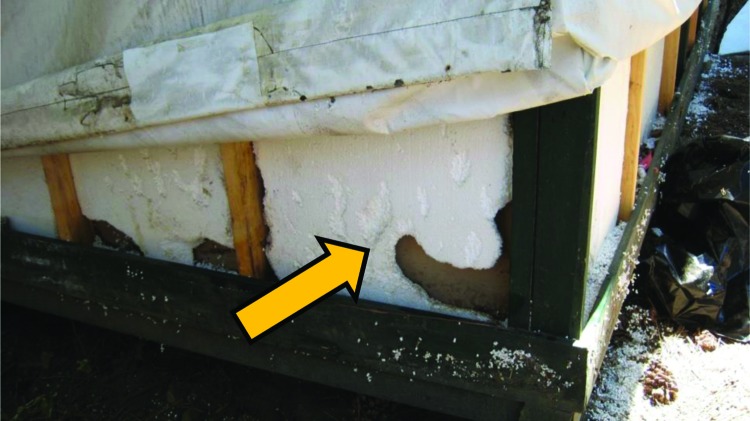
Damage from rodents tunneling in the foam insulation of a signature tent cabin, Yosemite National Park, summer 2012.

In August 2012, direct (e.g., feces) and indirect (e.g., gnaw holes) evidence of rodent activity was observed in both signature and regular tent cabins. Opportunities for mouse entry were also observed for both types of cabins. Regular tent cabins evaluated often had gaps between the door and threshold and gaps between the canvas and the frame or foundation. Most signature tent cabins evaluated had gaps between the door and threshold, gaps between the outer canvas tent and inner insulated wall, and holes in the exterior walls or floors, particularly around conduits for heaters. In late August 2012, when the cabins were being inspected and retrofitted to close these gaps, evidence of active rodent infestation (tunneling and nesting and live mice) was observed in the wall foam insulation of most signature tent cabins (Figure 4).

In the Tuolumne Meadows area, ≈8,725 feet above sea level, the regular tent cabins were similar to those in Curry Village: heavy canvas was affixed over a wood frame with either a wood or a cement stone, and stone foundation. Structural points of potential rodent entry were similar to those observed in Curry Village.

Rodent surveillance was conducted over 2 successive nights each in August and September 2012. On August 21–22, a total of 185 traps were placed around the signature tent cabins and other buildings in Curry Village. A total of 95 *Peromyscus* mice (trap success 51%) were collected: 73 *P. maniculatus* mice, 16 *P. boylii* mice, and 6 mice of *Peromyscus* species that could not be identified to species because raccoons had preyed on the mice in the traps. Serum antibodies against SNV were detected in 10 (14%) of 73 *P. maniculatus* and 0 of 16 *P. boylii* mice. On September 4–5, a total of 133 traps were placed in Curry Village, primarily in Boystown, and 89 traps were placed in Tuolumne Meadows. In Curry Village, 10 *P. maniculatus* mice and 9 *P. boylii* mice were collected (trap success 14%); no SNV serum antibodies were detected. In Tuolumne Meadows, 39 *Peromyscus* mice (all *P. maniculatus*), were collected (trap success 44%); SNV serum antibodies were detected in 2 (5%).

### Outreach and Prevention Efforts

During August 27–September 17, 2012, Yosemite contacted ≈10,000 guests who had stayed in signature tent cabins, ≈30,000 who had stayed in regular tent cabins, and ≈230,000 guests who had stayed in other park lodging, representing visitors from ≈77 different countries. The Yosemite public hotline received >4,800 calls. Hantavirus educational materials were handed to every park visitor, and educational messages were posted in public areas, in lodgings, and on the park Internet site. Additional hantavirus awareness and prevention training sessions were provided to employees and volunteers within the park and NPS-wide. CDC developed an Internet page specific to the outbreak and added staff to its toll-free hantavirus hotline.

On August 28, 2012, Yosemite closed all 91 signature tent cabins indefinitely when it was confirmed that 4 of the 6 patients identified at that time had lodged in 1 of the cabins and when mouse infestations in the cabin walls were noted. The cabins were subsequently dismantled. Approximately 1,300 buildings throughout the park were inspected, and rodent exclusion was performed where needed. Enhanced housekeeping, inspection, and documentation and response protocols were implemented. Additionally, park management began a rodent population surveillance program and implemented trapping in highly developed areas of Yosemite Valley. NPS-wide efforts include enhanced education and training opportunities, integration of public health review into building design and modification reviews, and enhanced guidelines for routine rodent exclusion inspections in all park structures.

## Discussion

The 2012 outbreak of hantavirus infections among Yosemite visitors was the largest recognized outbreak of SNV infections in the United States since the Four Corners outbreak in 1993. Ten persons had laboratory-confirmed SNV infection after lodging in Yosemite during June–July 2012. Eight had HPS and 3 died. In 1 patient, HPS had an incubation period of 7 weeks, longer than the 6-week upper range typically reported for HPS (*5*,*18*).

Staying overnight in a signature tent cabin in Yosemite’s Curry Village during June–August 2012 was significantly associated with SNV infection. While staying at Yosemite, case-patients had engaged in activities and behavior that were similar to those of their non-ill travel companions, and no common behavioral risk was identified. Despite extensive notification of all registered guests during the outbreak period, no cases were identified among persons who had lodged in regular tent cabins or elsewhere in Curry Village. No cases were identified among park employees, some of whom routinely entered signature tent cabins for housekeeping duties. No cases were identified among park visitors who had not stayed overnight.

This rare hantavirus outbreak was unusual because it was associated with exposure to rodents in a specific type of housing. Yosemite’s signature tent cabins were unique to the Boystown area in Curry Village and were not located elsewhere in Yosemite or in the NPS system. They were constructed in 2009 with dry wall and foam insulation to provide lodging for guests during winter. The presence of active deer mouse infestations within the insulated spaces of the cabins likely increased the risk for SNV exposure for persons staying overnight in these cabins. Although lodging in a signature tent cabin was a statistically significant risk factor for SNV infection, the overall incidence was small, occurring in ≈1 of 1,000 guests.

The 51% trap success for *Peromyscus* mice in Curry Village in August 2012 was substantially higher than the 11% (58 of 538) trap success in Yosemite Valley, including Curry Village, in April 1995 (CDPH, unpub. data) and 13%–21% trap success at ecologically comparable US Forest Service facilities in California in 2004–2005 (*19*). These data indicate that a robust population of deer mice was active at Curry Village in summer 2012. This population, however, appeared to be substantially lower in September (trap success 14%), after the signature tent cabins were closed in late August and rodent control measures were implemented. The presence of SNV (14% seroprevalence) among these deer mice was comparable to the 16% seroprevalence reported previously in deer mice trapped at similar elevations in California (*19*).

One case-patient had not stayed in a signature tent cabin or in Curry Village but had lodged in regular tent cabins in the Tuolumne Meadows area. Although this patient had visited Yosemite during the same period, his illness was unrelated epidemiologically to the signature tent cabin exposures linked to the other patients and represented a sporadic case of hantavirus infection. Two previous cases of HPS were identified in 2000 and 2010 among California residents who had visited the Tuolumne Meadows area during their illness incubation periods. Rodent surveillance conducted in September 2007 and September 2008 in Tuolumne Meadows documented *Peromyscus* mice trap successes of 14% and 17%—all *P. maniculatus* mice (CDPH, unpub. data). The 44% trap success in September 2012 indicated that the deer mouse population there was more robust than in previous years. However, SNV serum antibodies had been noted in ≈22% of deer mice in Tuolumne Meadows in 2007–2008 (CDPH, unpub. data), an appreciably higher percentage than the 5% observed during our investigation.

The 2012 Yosemite outbreak differed from the 1993 Four Corners outbreak in certain respects. The Yosemite outbreak occurred over a much shorter period; Yosemite patients were exposed and experienced illness over 1.5 months, whereas cases from the Four Corners outbreak occurred over 7 months (*2*–*5*). Also, Yosemite patients’ exposures occurred in a smaller geographic area and in a national park recreational area rather than private residential settings.

No specific treatment exists for HPS, but prompt recognition of symptoms and early initiation of supportive care can reduce death rates. In addition, extracorporeal membrane oxygenation might be helpful for infected patients who experience severe cardiopulmonary failure (*20*). Although HPS is rare and distinguishing its prodromal symptoms from other nonspecific viral syndromes is difficult, clinicians should consider HPS for all persons with febrile illness, sudden onset of respiratory symptoms, and progressive thrombocytopenia who have a history of possible rodent exposure. HPS remains a reportable disease in the United States, and all suspected cases should continue to be reported to the respective local or state health department for notification and confirmatory testing. Obtaining patient’s thorough travel history, which includes potential rodent exposures in rural settings (e.g., camping or opening seasonally closed buildings), can be useful for ascertaining the possibility of hantavirus infection.

Two case-patients in our study had milder disease that did not progress to HPS. Mild and subclinical illnesses are believed to represent a minor proportion of SNV infections (*21*,*22*). These 2 milder cases probably would not have been recognized if not for the publicity generated about the outbreak. A diagnosis of hantavirus infection was considered and pursued for both patients only after recovery and because of their report of having visited Yosemite during the weeks preceding their illnesses. These 2 cases demonstrate that mild SNV infections might be underdiagnosed, and thus, the 10 clinical cases identified in this investigation might underestimate the true incidence of SNV infection in this outbreak. Furthermore, media attention regarding this outbreak also led to the identification of 2 fatal cases; at the time of death for both patients, HPS was not suspected, and diagnostic testing was pursued only after media reports led clinicians to reconsider the cause of death.

The investigation possibly did not identify all cases of SNV infection among visitors to Yosemite during summer 2012. In addition, Yosemite visitors who had already recovered from a febrile illness at the time of notification might have been less motivated to again seek medical attention and be tested for SNV. Because HPS is a rare disease, hospitalized patients with unexplained acute respiratory distress syndrome might not have had HPS included in their differential diagnosis and therefore were not tested for hantavirus infection.

The observations at Curry Village made during the environmental investigation might not reflect conditions existing earlier, when the patients were exposed. The first environmental assessment was not conducted until ≈2 months after the earliest HPS patient had visited Yosemite. Nevertheless, the robust rodent population indicated by the high trap success in August 2012 and extensive evidence of deer mouse infestation of the signature tent cabins indicated that opportunity for exposure of guests to rodent excreta was likely high when the patients were staying there.

Yosemite management, in concert with local, state, and federal public health officials, took multiple actions to reduce the risk for hantavirus transmission to park visitors. Closing the signature tent cabins indefinitely, even before all patients were identified and all data were collected, was judicious and prevented more guests from being exposed. Regular tent cabins that replaced the dismantled signature tent cabins were designed and constructed with particular attention to rodent proofing. Continuous public awareness and rodent control and exclusion are key measures for minimizing the risk for hantavirus infection in areas inhabited by deer mice.
